# Case Report: A case series: heart rate variability during dental treatment with vasovagal reflex

**DOI:** 10.3389/fphys.2025.1607816

**Published:** 2025-07-16

**Authors:** Kaoru Yamashita, Toshiro Kibe, Shusei Yoshimine, Akari Uto, Minako Uchino, Mitsutaka Sugimura

**Affiliations:** ^1^ Department of Dental Anesthesiology, Field of Oral and Maxillofacial Rehabilitation, Advanced Therapeutics Course, Graduate School of Medical and Dental Sciences, Kagoshima University, Kagoshima, Japan; ^2^ Department of Oral and Maxillofacial Surgery, Field of Oral and Maxillofacial Rehabilitation, Advanced Therapeutics Course, Graduate School of Medical and Dental Sciences, Kagoshima University, Kagoshima, Japan

**Keywords:** autonomic nervous system activity, case series, dental treatment, heart rate variability, vasovagal reflex

## Abstract

**Introduction:**

The vasovagal reflex is the most frequent emergency that occurs during dental procedures, but its underlying mechanism is not understood. In this study, we conducted autonomic monitoring of patients with a history of vasovagal reflexes.

**Case description:**

We focused on the high-frequency component, an indicator of parasympathetic activity, and interrupted the treatment when the high-frequency component increased. Treatment was then resumed after confirming that there was no mood disturbance and no increase in the high-frequency component. In another patient with a history of dental treatment-induced vasovagal reflex, autonomic activity during treatment was measured under atropine sulfate hydrate administration.

**Discussion:**

Analysis of heart rate variability during the vasovagal reflex showed that parasympathetic hyperactivity was followed by sympathetic hyperactivity, indicating real-time changes in autonomic nervous system activity. In addition, the high-frequency component, which decreased after atropine sulfate hydrate administration, did not increase during treatment, along with the low-frequency to high-frequency ratio, a measure of sympathetic nervous system activity, and the vasovagal reflex did not occur. We believe that the visualization of a patient’s autonomic nervous system activity during dental treatment will improve the quality of systemic management and lead to the realization of a safe and comfortable treatment environment.

## 1 Introduction

Dental treatment is a medical procedure in which auditory stress, pain, anxiety, and tension cause fluctuations in autonomic nervous system (ANS) activity, which can lead to medical emergencies. Among these, the vasovagal reflex (VVR) is a frequent contingency (Obata et al., 2021), and analysis of ANS activity is necessary to establish a safe dental treatment environment. Heart rate variability (HRV) analysis is a method for analyzing ANS activity (Johansen et al., 1997; Lindh et al., 1997; Pagani et al., 1986; Sztajzel, 2004; Wakana et al., 2022; Esteban Pellicer et al., 2023; Pellicer et al., 2024). We previously analyzed HRV during dental treatment (Uchino et al., 2021; Uto et al., 2025; Yamashita et al., 2019; Yamashita et al., 2020a; Yamashita et al., 2020b; Yamashita et al., 2021; Yamashita et al., 2023; Yamashita et al., 2024). However, there are few reports on HRV analysis during VVR (Momota et al., 2014). In addition, the pathogenesis of VVR is largely unknown and data from HRV analysis would be useful in accumulating evidence on the development of VVR. We report our analysis of data from three patients with a history of VVR and discuss our findings.

## 2 Case description

### 2.1 Cases

Ethical approval was obtained from the Clinical Research Ethics Review Committee of Kagoshima University Hospital (No.: 230332 [case report]). Written informed consent was obtained from all patients. HRV analysis was performed with an electrocardiogram (MWM01; GMS) using MemCalc-Makin2 (GMS, Tokyo, Japan).

HRV represents the sinus node firing cycle variability, measured as the RR interval variability on electrocardiography and divided into components with spectral analysis. Frequencies between 0.04 Hz and 0.15 Hz are defined as the low frequency (LF) component and frequencies above 0.15 Hz as the high frequency (HF) component. The LF component reflects sympathetic and parasympathetic activity, while the HF component is derived from parasympathetic activity. Therefore, the ratio LF/HF indicates sympathetic activity.

Psychological testing was performed preoperatively using the Modified Dental Anxiety Scale (MDAS) and the State-Trait Anxiety Scale (STAI). The STAI-S (State Anxiety scale) assesses a transient situational response to “how I feel right now,” for an anxiety-provoking event, whereas the STAI-T (Trait Anxiety scale) assesses the tendency for a relatively stable response to an anxiety-provoking experience, such as “how I usually feel” in general. An MDAS score of ≥19 in a patient is associated with “strong” dental phobia, whereas an STAI-S score of ≥41 for men and ≥42 for women and an STAI-T score of ≥44 for men and ≥45 for women are associated with “high” anxiety.

### 2.2 Case 1

#### 2.2.1 Overview

The patient was a 23-year-old man (height 181 cm, weight 68 kg) with no history or genetic predisposition to syncope, who experienced VVR twice after local anesthesia in a dental clinic and was referred to our hospital for systemic management.

To reduce the patient’s anxiety, tooth extraction was planned under the supervision of the Department of Dental Anesthesiology. Sedation with nitrous oxide inhalation or an intravenous anesthetic was presented to the patient. However, the patient opted for a tooth extraction based on monitoring only due to concerns about drug administration. In addition to the patient’s blood pressure, heart rate, and oxygen saturation, the activity of the ANS was monitored by HRV analysis. Noninvasive blood pressure measurements were obtained every 2 min, and the low-frequency/high-frequency ratio (LF/HF) was calculated every 2 s using MemCalc-Makin2 (GMS).

#### 2.2.2 Progress in the case of occurrence of VVR

**FIGURE 1:**
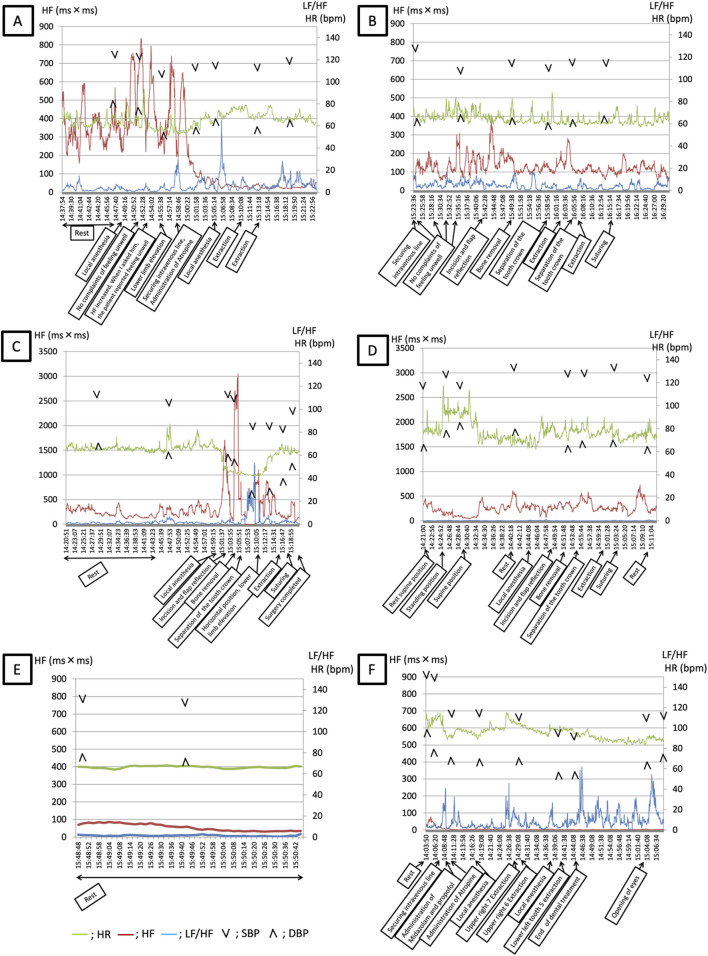
**(A,B)** Course of LF/HF, HF, HR, and SBP in Case 1. **(A)** Course at the onset of the vasovagal reflex: extraction of bilateral palatally displaced maxillary second premolars. **(B)** Course when the vasovagal reflex did not occur: extraction of the left mandibular third molar. Green line: HR, Red line: HF, Blue line: LF/HF, Arrowheads: BP LF/HF: Low frequency/High frequency; HF: High frequency; HR: Heart rate; SBP: Systolic blood pressure; DBP: Diastolic blood pressure. Timeline **(A)** 14:37–14:47 Rest 14:48 Local anesthesia 14:52 No complaints of feeling unwell 14:53 HF increased; when I asked him, the patient reported feeling unwell 14:55 Lower limb elevation 14:57 Securing intravenous line; administration of atropine 15:05 Local anesthesia 15:10 Extraction 15:13 Extraction. Timeline **(B)** 15:23 Securing an intravenous line 15:24–15:35 No complaints of feeling unwell 15:41 Incision and flap reflection 15:49 Bone removal 15:58 Separation of the tooth crown 16:02 Extraction 16:04 Separation of the tooth crown 16:07 Extraction 16:15 Suturing. **(C,D)** Course of LF/HF, HF, HR, and SBP in Case 2 **(A)** Course at the onset of the vasovagal reflex: extraction of the left mandibular third molar. **(B)** Course when the vasovagal reflex did not occur: extraction of the right mandibular third molar. Green line: HR, Red line: HF, Blue line: LF/HF, Arrowheads: BP LF/HF: Low frequency/High frequency; HF: High frequency; HR: Heart rate; SBP: Systolic blood pressure; DBP: Diastolic blood pressure Timeline **(C)** 14:20–14:43 Rest 15:00 Local anesthesia 15:02 Incision and flap reflection 15:04 Bone removal 15:05 Separation of the tooth crown 15:10 Horizontal position; lower limb elevation 15:15 Extraction 15:17 Suturing 15:20 Surgery completed Timeline **(D)** 14:21 Rest supine position 14:24 Standing position 14:29 Supine position 14:40 Rest 14:44 Local anesthesia 14:48 Incision and flap reflection 14:51 Bone removal 14:54 Separation of the tooth crown 15:00 Extraction 15:03 Suturing 15:10 Rest **(E)** Course of LF/HF, HF, HR, and SBP in Case 3 **(A)**. Records from the hospital room. **(B)**. Recording vital signs with history of a vasovagal reflex and prophylactic atropine administration during intravenous sedation. Green line: HR, Red line: HF, Blue line: LF/HF, Arrowheads: BP LF/HF: Low frequency/High frequency; HF: High frequency; HR: Heart rate; SBP: Systolic blood pressure; DBP: Diastolic blood pressure Timeline **(E)** 15:48–15:50 Rest Timeline **(F)** 14:03 Rest 14:06 Securing intravenous line 14:08 Administration of midazolam and propofol 14:10 Atropine administration 14:17 Local anesthesia 14:27 Upper right 7 extraction 14:29 Upper right 6 extraction 14:38 Local anesthesia 14:40 Lower left tooth 5 extraction 14:44 End of dental treatment 15:03 Opening of eyes.

The patient was scheduled for extraction of bilateral palatally displaced maxillary second premolars. Attempts were made to obtain the patient’s consent to secure an intravenous line, but the patient did not consent owing to anxiety. Table 1 shows the results of preoperative psychological testing. STAI-T scores indicating characteristic anxiety were classified as “high.” Figure 1A shows the results of blood pressure, heart rate, and HRV analyses: after administration of 1.3 mL of 2% lidocaine containing 1/80,000 adrenaline, the patient did not complain of discomfort, and no abnormalities were detected in vital signs. Subsequently, the high-frequency component of parasympathetic activity increased, and the patient complained of feeling unwell; pale face, cold sweat, and a decrease in heart rate were also observed.

**TABLE 1:** Cases 1 and 2: Psychological test results.

Case number	Psychological test	1st time (with vasovagal reflex)	2nd time (no vasovagal reflex)
Case 1	MDAS score	5	6
	STAI-S/STAI-T scores	23/60	26/62
Case 2	MDAS score	9	9
	STAI-S/STAI-T scores	39/69	46/62

MDAS, modified dental anxiety scale; STAI, State-Trait Anxiety Scale; STAI-S, state anxiety scale; STAI-T, trait anxiety scale.

Therefore, the lower extremities were elevated, and an intravenous line was secured; 0.5 mg of atropine sulfate hydrate was administered, the parasympathetic component decreased, and heart rate recovered.

The patient’s discomfort disappeared, and no further decrease in blood pressure or heart rate was observed. The patient’s symptoms dissipated, the blood pressure and heart rate normalized, and the procedure was resumed based on the criterion of recovery. After the dentist resumed treatment, the effects of atropine persisted, and the patient’s parasympathetic activity remained lower than that at the onset of VVR.

A cardiac cause for the syncope was ruled out based on the electrocardiographic findings, and although blood glucose levels were not measured, hypoglycemia was excluded based on the subsequent recovery. No side effects such as tachycardia or dry mouth due to atropine administration were observed.

#### 2.2.3 Progress in the case of non-occurrence of VVR

One month after the above procedure, the patient was scheduled for extraction of the left mandibular third molar owing to pericoronitis. Table 1 shows the results of preoperative psychological testing. Figure 1B shows the changes in blood pressure and heart rate, as well as the results of the HRV analysis.

There were no obvious changes in the MDAS, STAI-S, and STAI-T scores. As in the previous analysis, the patient had an STAI-T score classified as “high” and was administered 4 mL of 2% lidocaine containing 1/80,000 adrenaline in multiple small divided doses over time. The patient did not complain of discomfort and no abnormalities were noted in vital signs. Additionally, the high-frequency component was lower than that recorded at the time of the previous extraction. The procedure was stopped when the high-frequency component increased and the patient was asked if he was feeling well; after confirming that the high-frequency component had not increased, the procedure was resumed. Treatment was completed without any systemic complications. No VVR was observed within the following 6 months.

### 2.3 Case 2

#### 2.3.1 Overview

A 30-year-old female patient (height 165 cm, weight 54 kg) with a phobia of dental treatment was referred to our university hospital for bilateral mandibular third molar caries requiring extraction. The same monitoring was performed as that in case 1.

#### 2.3.2 Progress in the case of occurrence of VVR

The patient was scheduled for extraction of a left mandibular wisdom tooth with caries. Table 1 shows the results of preoperative psychological testing. The STAI-T score, indicating characteristic anxiety, was classified as “high.” Figure 1C shows the results of blood pressure, heart rate, and HRV analyses. A sharp increase in the high-frequency component and decrease in blood pressure and heart rate were observed during bone removal and tooth crown separation, and the patient complained of feeling unwell. Thereafter, the LF/HF increased, and the patient’s sympathetic tone and heart rate further increased upon positional change from supine to lower extremity elevation.

The patient’s symptom resolution and normalization of blood pressure and heart rate were considered signs of recovery, and the procedure was continued.

Treatment was completed without any complication. A cardiac cause for the syncope was ruled out based on the electrocardiographic findings, and although blood glucose levels were not measured, hypoglycemia was excluded based on the subsequent recovery. No side effects such as tachycardia or dry mouth due to atropine administration were observed.

#### 2.3.3 Progress in the case of non-occurrence of VVR

The patient had a planned extraction of a right mandibular impacted wisdom tooth with caries. Table 1 shows the results of preoperative psychological testing. The MDAS score had not changed since the previous extraction. The STAI-S score increased and was classified as “high,” and the STAI-T decreased but was classified as “high.” Figure 1D shows the results of blood pressure, heart rate, and HRV analyses. The patient did not complain of feeling unwell and no abnormal vital signs were observed. Treatment was successfully completed; the LF/HF was lower than that recorded at the previous extraction. No VVR was observed within the following 6 months.

### 2.4 Case 3

#### 2.4.1 Overview

The patient was a 55-year-old woman (height 162.2 cm, weight 50.8 kg) who had three episodes of VVR after local anesthesia. She experienced seizures, nausea, loss of consciousness, rigidity of extremities, and incontinence during the first episode. Even in the second episode, she presented to the emergency department with loss of consciousness, incontinence, and persistent vomiting, and was diagnosed with VVR. During the third episode, she also experienced loss of consciousness and incontinence. The patient had experienced similar symptoms once every few years and had no history of VVR when an intravenous line was secured but she had a fear of dental procedures. Therefore, the patient was admitted to the hospital, and treatment consisted of monitoring and intravenous sedation, as in case 1.

#### 2.4.2 Progress in the case of non-occurrence of VVR

The patient was scheduled for extraction of the right maxillary first and second M and the left mandibular second premolar. Table 2 shows the results of the preoperative psychological tests. The patient was classified as phobic of dental treatment because of an MDAS score of ≥19. The STAI-S and STAI-T scores were classified as “high.” Figures 1E,F show the trends in blood pressure, heart rate, and HRV at rest the previous day and during the procedure under intravenous sedation, respectively. Atropine 0.5 mg was administered intravenously 5 min before trigeminal nerve stimulation. As a result of prophylactic atropine administration, the intraoperative high-frequency component was low, and no increase in the high-frequency component was observed, even when the LF/HF increased (Figure 1F). Compared with those in the resting state (Figure 1E), the heart rate and LF/HF increased and the high-frequency component decreased. The patient did not develop VVR during or after treatment. No VVR was observed within the following 6 months.

**TABLE 2:** Case 3: Psychological test results.

Psychological test	Atropine administration
MDAS score	24
STAI-S/STAI-T scores	43/54

MDAS ≥19 strong dental phobia.

STAI-S ≥41 (Men), STAI-S ≥42 (Women) High anxiety.

STAI-T ≥44 (Men), STAI-T ≥45 (Women) High anxiety.

MDAS, modified dental anxiety scale; STAI, State-Trait Anxiety Scale; STAI-S, state anxiety scale; STAI-T, trait anxiety scale.

## 3 Discussion

**FIGURE 2:**
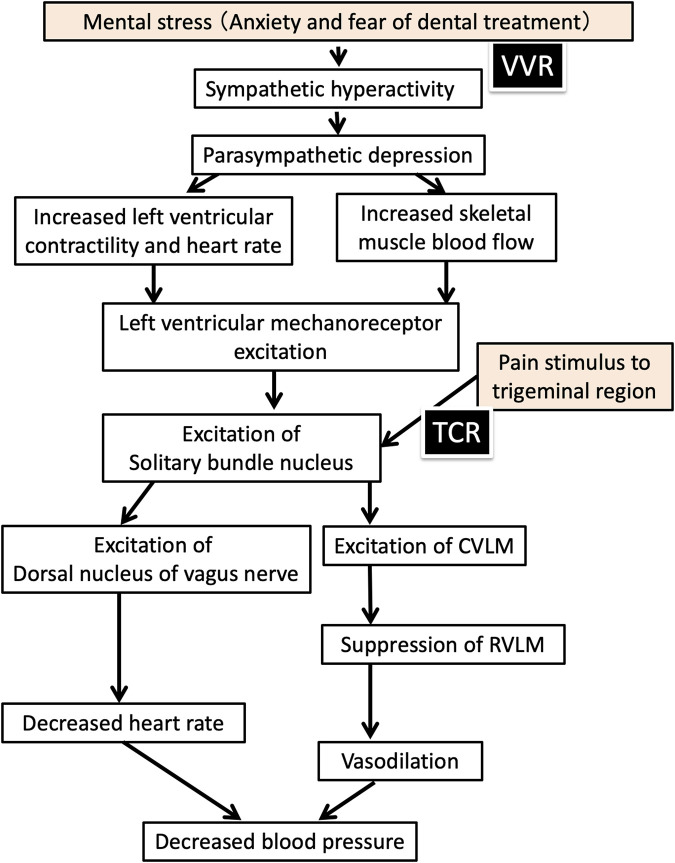
Mechanism of vasovagal reflex and trigeminal cardiac reflex in dental treatment (modified from Figure 13-II-1 of Fukushima Kazuaki et al. Dental Anesthesiology, eighth Edition, ISHIYAKU publishers, 2019; 507.) VVR: Vasovagal reflex; TCR: Trigeminal cardiac reflex; CVLM: Caudal ventrolateral medulla; RVLM: Rostral ventrolateral medulla.

Using HRV analysis to measure ANS activity in three patients with a history of VVR, we were able to capture changes at the onset of VVR in real time in cases 1 and 2. The mechanism of VVR development can be divided into two pathways: 1) the pathway of sympathetic nervous activation increases left ventricular contractility, heart rate, and skeletal muscle blood flow, excites left ventricular mechanoreceptors, and transmits stimulation to the nucleus tractus solitarii, causing a decrease in heart rate and blood pressure (Niwa et al., 1996); and 2) stimulation of the peripheral branches of the trigeminal nerve excites the dorsal nucleus of the vagus nerve via interneurons from the trigeminal spinal tract nucleus (Meuwly et al., 2015), a possible pathway to induce parasympathomimetic symptoms (Figure 2). In cases 1 and 2, the increase in the high-frequency component preceded the increase in the LF/HF. Therefore, the involvement of pathway (2) in the pathogenesis of the trigeminal cardiac reflex (TCR) was considered (Blanc et al., 1983; Meuwly et al., 2015; Meuwly et al., 2017; Niwa et al., 1996; Raj, 2008), and we hypothesized that VVR could be prevented by monitoring the increase in the high-frequency component during dental treatment that stimulates the trigeminal innervated area. We monitored the high-frequency component, an index of parasympathetic activity, during the second treatment in cases 1 and 2. When the high-frequency component increased, the treatment was interrupted, and the patient was asked if he or she was feeling unwell. After confirming that there were no problems, the surgery was resumed, and safe management was possible through HRV monitoring.

In case 3, we analyzed HRV in a patient suspected of having a history of TCR and performed dental treatment under atropine premedication (Rama et al., 1997). Many reports have shown the efficacy of atropine in preventing bradycardia owing to TCR (Wang et al., 2022; Yoshida et al., 2022). Lübbers et al. (2010) recommended the prophylactic administration of 0.5 mg of intravenous atropine immediately before surgical procedures that may induce TCR. Therefore, 0.5 mg of atropine was administered via the intravenous route before surgery, and surgery could be performed without TCR.^12^ This suggests that the suppression of parasympathetic nervous system (PNS) activity after atropine administration contributes to the prevention of TCR by suppressing the increase in PNS activity that follows the increase in sympathetic nervous system (SNS) activity during dental treatment. In addition, he was classified as phobic of dental treatment according to the MDAS score, which was considered an indication for intravenous sedation. As with local anesthesia, intravenous access involving needle insertion can lead to VVR. However, this patient had no history of VVR caused by venipuncture. Therefore, we chose to manage the patient with intravenous sedation.

TCR is preceded by increased PNS activity due to nociceptive stimulation to the trigeminal nerve sensory branch, whereas VVR is preceded by increased SNS activity due to psychological stress. Therefore, ANS activity monitoring by HRV analysis is expected to be useful in differentiating between TCR and VVR. In VVR, which is preceded by increased SNS activity, an approach to anxiety and tension is important, whereas in TCR, an approach to the PNS activity itself is necessary. ANS activity monitoring is also useful in the selection of management methods, such as music listening and psychosedation, to reduce mental tension in patients with VVR. Premedication with atropine, a parasympathetic nerve blocker, can also prevent TCR. HRV analysis during dental treatment would have been useful because of the clear association between HRV trends and autonomic pathways. Furthermore, the patients said, “monitoring autonomic nervous activity gave me a sense of security,” “I was able to receive treatment without feeling fear like before,” and “I would like to have autonomic nervous activity monitored if I receive treatment again.” However, further research is needed to determine the relationship between the scores and development of VVR. In addition, data on HRV analysis during dental treatment incidents can be obtained incidentally, and few relevant cases have been reported. Therefore, it is necessary to conduct multicenter collaborative studies to accumulate data.

Three patients were satisfied with the autonomic nerve monitoring management, saying that they were able to receive treatment with peace of mind.

A limitation of this study was that there were only three cases. More cases will be analyzed in the future.

## 4 Conclusion

HRV analysis allowed us to capture the ANS activity of TCR that developed during dental treatment in real time. Monitoring the increase in high-frequency components that stimulate the trigeminal nerve area during dental treatment is useful for predicting TCR. In addition, atropine administration contributes to the prevention of the development of TCR by inhibiting the increase in PNS activity following the increase in SNS activity during dental procedures. Analysis of HRV during dental treatment could lead to the creation of a safe dental treatment environment.

## Data Availability

The original contributions presented in the study are included in the article/supplementary material, further inquiries can be directed to the corresponding author.
